# Controllable Preparation of Large-Area Ordered MoS_2_ Nanotube Arrays with Enhanced Optoelectronic Detection Performance

**DOI:** 10.3390/nano16110678

**Published:** 2026-05-29

**Authors:** Haowei Lin, Mingxuan Li, Wenbo Chen, Jing Chen, Hao Cai, Li Li, Mengdan Li, Yuhang Pan

**Affiliations:** School of Materials Science and Engineering, Henan University of Technology, Zhengzhou 450001, China; limingxuan0722@163.com (M.L.); cwb2047975933@163.com (W.C.); chenqingmail2024@163.com (J.C.); 18771662994@163.com (H.C.); li_li200404@163.com (L.L.); lmd041025@163.com (M.L.); 18800729686@163.com (Y.P.)

**Keywords:** MoS_2_, nanotube arrays, controlled synthesis, optoelectronic detection performance

## Abstract

Large-area MoS_2_ nanotube arrays were successfully prepared using a combination of simple and reliable electrochemical deposition and chemical etching techniques, with highly ordered ZnO nanorod arrays used as the template. The thickness of MoS_2_ nanotube walls can be effectively controlled by adjusting the deposition time. The characterization results of SEM and TEM showed the successful preparation of MoS_2_ nanotube arrays with different wall thicknesses. The composition of the obtained nanotube arrays was verified to be MoS_2_ by EDS, XRD, and XPS characterizations. It is worth noting that compared to MoS_2_ nanofilms, the as-prepared MoS_2_ nanotube arrays exhibit stronger photoelectric response properties; the on/off ratio and photoresponsivity increased by 2.8 times and 3.8 times, respectively, mainly attributed to its significantly increased specific surface area. These research results provide new ideas for the large-area controllable preparation of MoS_2_ low-dimensional nanostructures, as well as new material candidates for the development of low-cost and high-performance photodetectors.

## 1. Introduction

Molybdenum disulfide (MoS_2_), as a typical two-dimensional layered transition metal sulfide compound, has become a highly promising optoelectronic detection material due to its atomically flat interface, layer-dependent adjustable bandgap (direct bandgap ~1.8 eV for a single layer), and high light absorption coefficient and carrier mobility. MoS_2_-based photodetectors exhibit a wide spectral response capability, from ultraviolet to near-infrared, and in recent years, their core indicators, such as responsivity, response speed, and detection rate, have been continuously refreshed [1,2,3,4,5,6,7,8]. However, the carrier capture effect caused by intrinsic defects and interface states has long constrained the synergistic improvement of response speed and responsiveness. In response to these challenges, researchers have significantly improved the performance of MoS_2_-based photodetectors through strategies such as heterostructure engineering, interface control, and plasmon enhancement [9,10,11,12,13,14,15,16,17]. On the other hand, researchers are attempting to improve the performance of optoelectronic detection devices from a perspective of material morphology. Compared to two-dimensional continuous thin films, one-dimensional structures not only retain a tunable bandgap and excellent light absorption ability of MoS_2_, but also introduce a higher specific surface area, stronger optical confinement effect, and axial oriented carrier transport channel [18]. These characteristics provide a new dimension for further improving photoelectric response performance and breaking the contradiction between response speed and responsiveness in traditional photodetectors. However, there are currently few reports on the controllable preparation and optoelectronic properties of large-area one-dimensional MoS_2_ nanostructures.

This paper uses highly ordered ZnO nanorod arrays as templates to achieve a controllable preparation of thickness-adjustable MoS_2_ nanotube arrays through a combination of electrochemical deposition and chemical etching techniques. As a semiconductor material with excellent optoelectronic properties, the preparation of low-dimensional nanostructures of MoS_2_ has been reported, such as the synthesis of MoS_2_ nanotubes via the hydrothermal method [19], the decoration of MoS_2_ on ZnO nanorods [20], and using carbon nanotubes as templates to prepare MoS_2_ nanotubes [21]. These studies have provided an important foundation for the construction of MoS_2_ nanostructures. In comparison with previous studies, our technical approach effectively integrates three key steps: template construction, deposition of target materials, and template removal, forming a coherent and concise preparation process. The main differences between our method and previously reported preparation techniques lie in its high degree of method integration, simplicity of process flow, and easier implementation of large-scale array preparation. Compared to mainstream preparation methods for MoS_2_ nanostructures [22,23,24], such as the hydrothermal, chemical vapor deposition (CVD), atomic layer deposition (ALD), or sulfide-based methods, our electrochemical deposition method cleverly combines multiple advantages, such as room temperature operation, controllable morphology, no need for high-temperature post-treatment, and simple equipment construction, opening up a new path for the preparation of MoS_2_ low-dimensional nanomaterials and providing a feasible solution to the engineering problem of how to prepare high-performance large-area nanoarrays simply, efficiently, and controllably. At the same time, the method exhibits obvious characteristics in terms of “economic applicability” and “process friendliness” and also provides an important foundation for the industrial application of MoS_2_. The photoelectric responsiveness of MoS_2_ nanotube arrays with different wall thicknesses is compared and analyzed. Research has shown that by adjusting the electrochemical deposition time (1 min, 2 min, 4 min, 6 min, 8 min, respectively), the wall thickness of MoS_2_ nanotubes can be effectively controlled (from 9.3 nm to 43.5 nm). The MoS_2_ nanotube arrays prepared by electrochemical deposition for 4 min exhibit the strongest photoelectric response performance, with a switching ratio of 25.31 and a photoelectric response rate of 0.29 mA/W. Compared to MoS_2_ thin films, the switching ratio and photoresponsivity of MoS_2_ nanotube arrays have increased by 2.8 times and 3.8 times, respectively. These research results provide new ideas for a large-area controllable preparation of MoS_2_ nanostructures and also provide useful references for the construction of low-cost and high-performance MoS_2_-based photodetectors.

## 2. Materials and Methods

### 2.1. Materials

Zinc nitrate hexahydrate, zinc acetate dihydrate, lithium hydroxide, hexamethylenetetramine, ammonium tetrathiomolybdate, nitric acid, and potassium chloride were purchased from Shanghai Aladdin Bio-Chem Technology Co., Ltd. (Shanghai, China). These reagents were used directly as purchased. Indium tin oxide (ITO) glass was purchased from Beijing Zhongjing Scientific Instrument Co., Ltd. (Beijing, China).

### 2.2. Synthesis of MoS_2_ Nanotube Arrays

**Figure 1:**
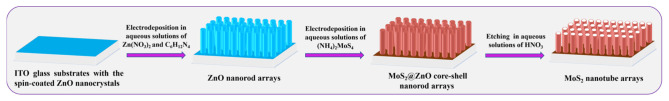
Schematic illustration of controlled synthesis of oriented MoS_2_ nanotube arrays.

The preparation process of MoS_2_ nanotube arrays is shown in Figure 1. Firstly, large-area ordered ZnO nanorod arrays were synthesized on an ITO glass substrate in a homemade glass cell at 90 °C in aqueous solutions of zinc nitrate hexahydrate (0.1 M) and hexamethylenetetramine (0.1 M) by using the electrochemical deposition method, which has been described in our previously published article [25]. Then, MoS_2_ was synthesized on the surface of ZnO nanorod arrays to prepare MoS_2_@ZnO core–shell nanorod arrays through electrochemical deposition, which was carried out in aqueous solutions of ammonium tetrathiomolybdate (0.1 M) and potassium chloride (0.05 M). This reaction relied on a three-electrode system. The ITO glass substrate with ZnO nanorod arrays, a platinum plate, and a saturated calomel electrode were used as the working electrode, the counter electrode, and the reference electrode, respectively. The electrodeposition reaction of MoS_2_ was conducted at room temperature and a constant voltage of −1.2 V, with a focus on examining the impact of reaction time (1 min, 2 min, 4 min, 6 min, and 8 min) on the thickness of the MoS_2_ shell layer. Finally, the obtained core–shell nanorod arrays were soaked in 0.01 M nitric acid aqueous solution for 1 h to dissolve the ZnO cores, resulting in the ordered MoS_2_ nanotube arrays.

**Figure 2:**
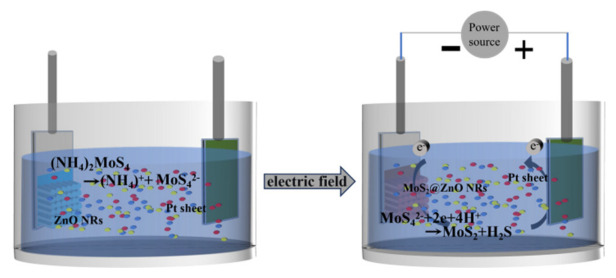
Schematic diagram of the deposition process of MoS_2_ on the surface of ZnO nanorod arrays.

As shown in Figure 2, when a voltage of −1.2 V is applied to the ZnO nanorod array, MoS_2_ is gradually deposited on the surface of the ZnO nanorods, with the core of [MoS_4_]^2−^ undergoing a reduction reaction to generate MoS_2_ [26]. The electrochemical deposition reaction can be expressed as:

(NH_4_)_2_MoS_4_ → 2NH_4_^+^ + MoS_4_^2−^

MoS_4_^2−^ + 2e^−^ + 4H^+^ → MoS_2_ + 2H_2_S

The electrodeposition of MoS_2_ on the surface of ZnO nanorods is a synergistic process of heterogeneous nucleation and layered growth, determined by the substrate surface chemical properties and electrochemical driving forces. There are naturally numerous defects on the surface of ZnO nanorods, including oxygen vacancies, zinc gaps, and surface dangling bonds. These defect sites have higher surface energy and stronger adsorption activity. When MoS_2_ is reduced and deposited from the solution phase, [MoS_4_]^2−^ ions will preferentially adsorb on these high-surface-energy defect sites, causing local supersaturation and inducing the formation of MoS_2_ crystal nuclei. Furthermore, there is a lattice mismatch of approximately 2.8% between the wurtzite structure of ZnO and the hexagonal layered structure of MoS_2_. This moderate mismatch induces the growth of MoS_2_ along a specific crystallographic orientation on the surface of ZnO, ultimately forming a uniform MoS_2_ shell layer [27].

### 2.3. Characterization

Field scanning electron microscopy (SEM) (FEI Inspect F50, Hillsborough, OR, USA) and transmission electron microscopy (TEM) (JEOL JEM-2010, Tokyo, Japan) were used to characterize the microstructures of the MoS_2_ nanotube arrays. The structural properties were extracted using an X-ray diffractometer (Bruker D8-Advance, Billerica, MA, USA) with a Cu-LFF (λ = 1.54 Å) tube operated at 40 kV–40 mA, employing a scanning rate of 5°/min in the 2θ range from 20° to 80°. Spectral properties were tested by a UV–visible spectrophotometer (Shimadzu UV-2600i, Tokyo, Japan). The composition of MoS_2_ nanotube arrays was tested using Energy Dispersive X-ray (Bruke XFlash 6130, Berlin, Germany) and X-ray Photoelectron Spectroscopy (Thermo Scientific K-Alpha, Waltham, MA, USA). The photoelectric performance of MoS_2_ nanotube arrays was tested using a semiconductor characteristic analyzer (Keithley 4200A-SCS, Cleveland, OH, USA), with the assistance of a xenon light source (PLS-SXE300, Beijing, China) providing an illumination intensity of 100 mW/cm^2^.

## 3. Results

**Figure 3:**
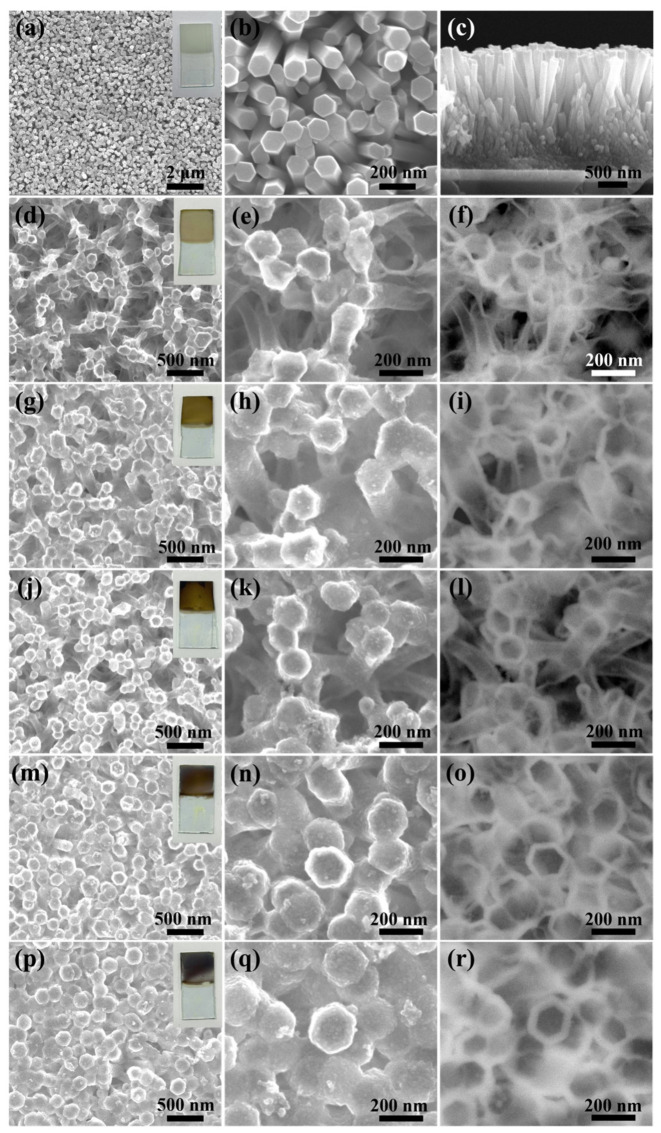
SEM images of ZnO nanorod arrays (**a**–**c**) and MoS_2_ nanotube arrays prepared under different reaction times: (**d**–**f**) 1 min, (**g**–**i**) 2 min, (**j**–**l**) 4 min, (**m**–**o**) 6 min, (**p**–**r**) and 8 min. (**f**,**i**,**l**,**o**,**r**) is the background scattered electron images corresponding to (**e**,**h**,**k**,**n**,**q**). The insets are the digital images of ZnO and MoS_2_ nanoarrays.

Using ordered ZnO nanorod arrays as the template, large-area MoS_2_ nanotube arrays were controllably synthesized through a strategy combining electrochemical deposition and chemical etching. Their microstructures were first investigated using scanning electron microscopy, and the results are shown in Figure 3. Figure 3a–c shows the top and side views of large-area ordered ZnO nanorod arrays prepared on ITO glass substrate. These nanorods exhibit a typical hexagonal shape with a relatively uniform diameter and length, approximately 140 nm and 1.9 μm, respectively. Such an ordered structure provides a good foundation for the preparation of large-area MoS_2_ nanotube arrays. Figure 3d–f illustrates the microstructure of the MoS_2_ nanotube arrays obtained after chemically etching the ZnO core. The nanotube walls are intact, and the surface is smooth, indicating that MoS_2_ formed a complete and uniformly thick shell structure on the surface of ZnO nanorods during the electrochemical deposition process. It is noteworthy that Figure 3c demonstrates the corresponding background scattered electron images of Figure 3d, where a typical nanotube-like structure can be clearly observed, indicating that the ZnO core was successfully dissolved during the etching process. Figure 3g–r displays the influence of electrochemical deposition time on the microstructure of MoS_2_ nanotube arrays. Through comparison, it can be observed that as the electrochemical deposition time increases, the thicknesses of the MoS_2_ nanotube walls significantly increase, and the adhesion between them gradually becomes more pronounced. As seen in the digital images of the ZnO and MoS_2_ nanoarrays in the insets, compared to the white ZnO nanorod arrays, the MoS_2_ nanotube arrays exhibit a gradually increasing brown color with prolonged electrochemical deposition time, and the overall color is relatively uniform, indicating successful preparation of large-area ordered MoS_2_ nanotube arrays.

**Figure 4:**
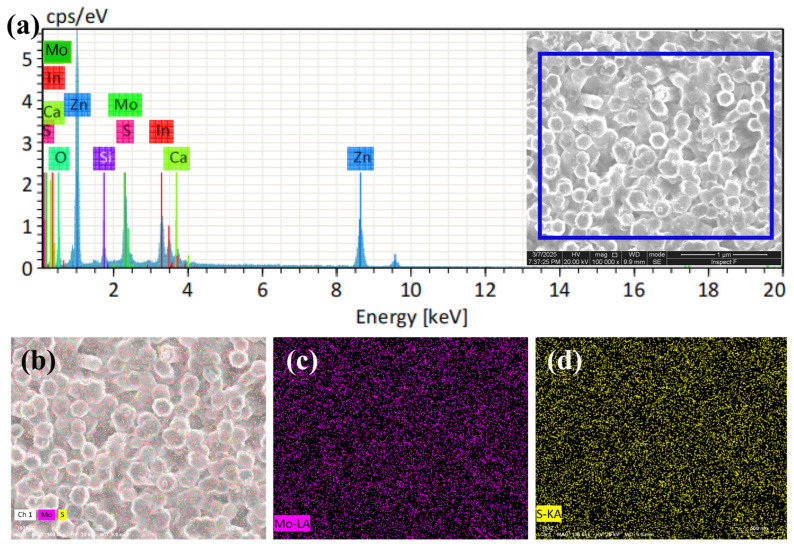
Energy dispersive X-ray spectroscopy (EDS) of MoS_2_ nanotube arrays: (**a**) selection analysis result displaying element types, (**b**) combined element mapping, (**c**) mapping of element M_O_, and (**d**) mapping of element S.

The elemental composition of the MoS_2_ nanotube arrays with an electrochemical deposition time of 6 min was qualitatively and quantitatively studied through EDS analysis. As shown in Figure 4a, the obtained nanotube arrays contain the characteristic elements Mo and S. The atomic ratio of Mo to S is about 1:2.3, which deviates from the theoretical value to some extent. This is mainly due to the high overlap of the characteristic peaks of Mo and S elements in the EDS spectrum, which leads to deviations in quantitative calculations. This phenomenon can also be seen in other reports [28]. The results also showed a certain amount of Zn and O elements, which is due to the MoS_2_ nanotube arrays being prepared on a layer of ZnO nanoparticles formed by spin coating, and it will not be damaged during the etching process of the ZnO nanorod core. In addition, In, Ca, and Si elements have also appeared, which originate from ITO glass substrates. Figure 4b–d presents the element mapping results of the MoS_2_ nanotube arrays, further verifying the distribution of Mo and S elements. The Mo and S elements are uniformly distributed in the obtained arrays and exhibit a certain coexistence relationship, and their distribution probability corresponds clearly to the morphology of the nanotubes in the SEM image. These results of selected analyses and elemental mapping collectively demonstrate the successful synthesis of MoS_2_ nanotube arrays.

**Figure 5:**
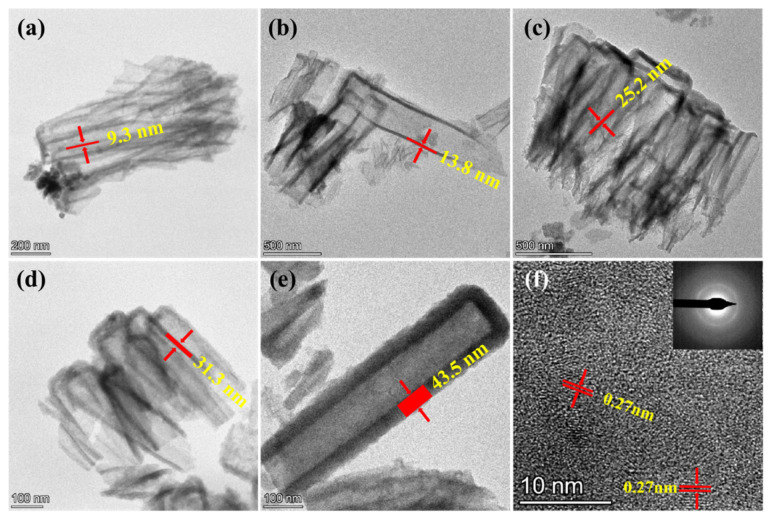
TEM images of MoS_2_ nanotube arrays prepared at different reaction times: (**a**) 1 min, (**b**) 2 min, (**c**) 4 min, (**d**) 6 min, (**e**) 8 min, and (**f**) 4 min. The inset is the selective area electron diffraction pattern (SAED) taken from the nanotube.

The internal structural characteristics of the MoS_2_ nanotube arrays were further characterized using TEM. Figure 5 shows the TEM image and selective area electron diffraction pattern (SAED) of the MoS_2_ nanotube array. Figure 5a–e displays TEM images of the MoS_2_ nanotubes at different deposition times of 1 min, 2 min, 4 min, 6 min, and 8 min, respectively. In Figure 5a, the image contrast of the MoS_2_ nanostructures exhibits a significant gradient distribution characteristic, and the interior of these nanostructures presents a typical hollow structure. The degree of contrast between the tube wall and the internal region is obvious, and the tube wall is relatively intact and presents a relatively uniform thickness, indicating the formation of MoS_2_ nanotube structures. As the electrochemical deposition time increases, we can observe that the wall thicknesses of the nanotubes gradually increase in Figure 5b–e, which are 9.3 nm, 13.8 nm, 25.2 nm, 31.3 nm, and 43.5 nm, respectively. This trend is consistent with the corresponding SEM image results. From the high-resolution TEM image in Figure 5f, it can be observed that the lattice spacing of MoS_2_ nanotubes is 0.27 nm, which matches the spacing between MoS_2_ (100) crystal planes [29], further verifying the successful synthesis of MoS_2_ nanotube arrays. In addition, by analyzing the selective area electron diffraction pattern (SAED), multiple concentric circular diffraction patterns were observed, indicating that the prepared MoS_2_ nanotube arrays have polycrystalline properties.

**Figure 6:**
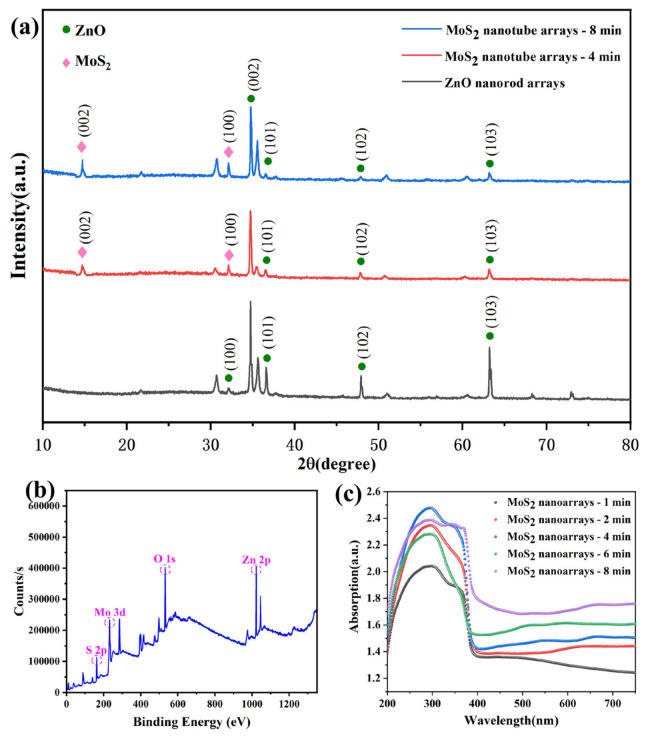
(**a**) XRD patterns of MoS_2_ nanotube arrays and ZnO nanorod arrays; (**b**) XPS pattern of MoS_2_ nanotube arrays; (**c**) normalized UV–visible absorption spectra of the different MoS_2_ nanotube arrays.

As shown in Figure 6a, the diffraction characteristics of the MoS_2_ nanotube arrays at different deposition times can be observed from the XRD pattern. At the electrochemical deposition time of 4 min, a distinct characteristic diffraction peak appeared in the XRD pattern of the MoS_2_ nanotube array, located at approximately 14.6°, corresponding to the (002) crystal plane of the MoS_2_ crystal. In addition, a strong diffraction peak can also be observed at 32°, representing the (100) crystal plane of MoS_2_ [30]. The positions of these diffraction peaks are consistent with the values of the standard card JCPDS, which verifies the composition of MoS_2_ nanotubes and also indicates their good crystalline quality. When the deposition time of MoS_2_ is extended to 8 min, the thicknesses of the MoS_2_ nanotubes increase, resulting in a significant increase in the intensity of MoS_2_ characteristic diffraction peaks at 14.6°and 32°, while the characteristic diffraction peaks of the substrate ZnO nanorods show a significant weakening trend. Figure 6b shows the XPS pattern of MoS_2_ nanotube arrays. The main peaks, appearing at 232.3 eV and 162.7 eV, are attributed to the binding energies of the 3d orbitals of Mo and the 2p orbitals of S, indicating that Mo and S elements exist in the form of Mo^4+^ and S^2−^, respectively [31]. Through quantitative calculation, the elemental contents of Mo and S are 14.02% and 24.06%, respectively, and their elemental ratios are highly consistent with the theoretical value of 1:2, which also confirms that the composition of the nanotubes is MoS_2_. The main peaks appearing at 1022.5 eV and 531.6 eV, respectively, belong to the binding energies of the 2p orbitals of element Zn and the 1s orbitals of element O, originating from the zinc oxide particle layer on the ITO glass substrate. Figure 6c shows the spectral absorption characteristics of MoS_2_ nanotube arrays in the wavelength range of 200–750 nm at different deposition times. There is a clear dual-band absorption characteristic of ultraviolet and visible light, and with the increase in deposition time, the absorption of MoS_2_ nanotube arrays in the visible light region gradually increases. This can be attributed to the gradual improvement of the MoS_2_ nanotube structure and the increase in volume, which has improved the light capture efficiency.

**Figure 7:**
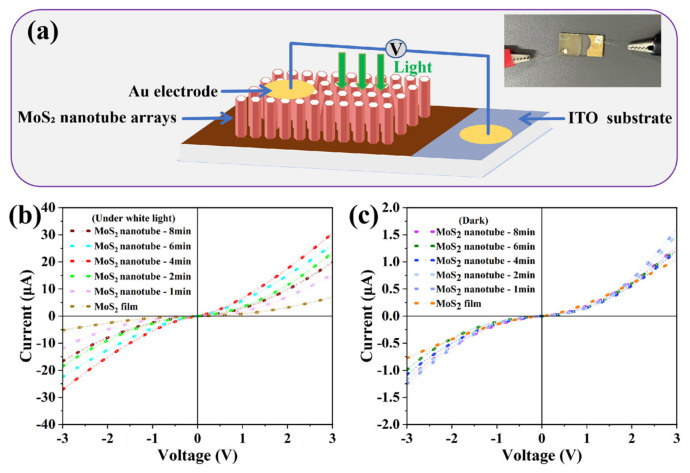
(**a**) Working model of MoS_2_ nanotube array device; (**b**) typical *I–V* curves of MoS_2_ nanotube arrays and MoS_2_ nanofilm under illumination of white light; (**c**) typical *I–V* curves of MoS_2_ nanotube arrays and MoS_2_ nanofilm in dark.

The photoelectric response behavior of the MoS_2_ nanotube arrays at different deposition times was compared and analyzed using the MoS_2_ nanofilm obtained by electrochemical deposition on ITO glass as reference materials, which was investigated by measurements of the current (I) versus bias voltage (V). The devices were fabricated as described in Figure 7a. The digital photo of this device is shown in the upper right corner of Figure 7a. Figure 7b and Figure 7c show the *I–V* characteristic curves of the MoS_2_ nanotube array and MoS_2_ nanofilm under illumination of white light and in the dark, respectively. It can be seen from the figure that when the bias voltage is 3 V, the current of the MoS_2_ nanofilm is 7.06 μA, the dark current is 1.05 μA, the on/off ratio is 6.72, and the photoelectric responsivity is 0.06 mA/W. The current of the MoS_2_ nanotube arrays (deposited for 1 min) is 15.42 μA, the dark current is 1.62 μA, the on/off ratio is 9.52, and the photoelectric responsivity is 0.14 mA/W. It can be seen that the on/off ratio and photoresponsivity of the MoS_2_ nanotube arrays are relatively improved compared to the MoS_2_ nanofilm. This is mainly due to the fact that when MoS_2_ is prepared as nanotube arrays, on the one hand, its specific surface area is significantly increased compared to the film, which can provide more light absorption and active sites. On the other hand, the orderly arrangement of nanotubes forms a fast transport channel, which is conducive to the orientation and long-distance migration of charge carriers. In addition, the unique hollow structure of nanotubes constitutes a microscopic “light trap”, which causes multiple reflections, refractions, and diffractions of incident light inside the tube, significantly increasing the interaction path between light and materials. The synergistic effect of these factors achieves an overall qualitative change in steps such as light absorption, charge separation, and transport, ultimately resulting in enhanced optoelectronic response performance [32].

As the growth time of the MoS_2_ nanotube arrays increases, the current shows a trend of first increasing and then decreasing. When the growth time is 4 min, the current increases to a maximum of 30.62 μA, the dark current is 1.21 μA, the on/off ratio is 25.31, and the photoelectric responsivity is 0.29 mA/W. The specific detectivity (D*) of the MoS_2_ nanotube arrays is 4.66 × 10^8^ Jones, which is calculated as follows:

$$D^{*}=R\sqrt{\frac{S}{2qI_{dark}}}$$

where *q* is the elemental charge of performance [33]. Compared to the MoS_2_ nanofilm, the on/off ratio and photoelectric responsivity of the MoS_2_ nanotube arrays increased by 2.77 times and 3.83 times, respectively. Subsequently, as the growth time prolongs, the photocurrent decreases slightly. This is closely related to the further improvement of the MoS_2_ nanotube structure with the extension of reaction time, and the corresponding SEM and TEM images can also clearly show that the nanotube structures are becoming more perfect. But when the reaction time exceeds 4 min, due to the significant increase in nanotube thickness and adhesion, the lifetime of photoelectrons is shortened. In addition, the nanoscale effect is reduced, resulting in a decrease in photocurrent.

## 4. Conclusions

In summary, this work presents a simple and reliable method for controllable synthesis of large-area ordered MoS_2_ nanotube arrays on ITO glass substrates. By adjusting the electrochemical deposition time, the wall thicknesses of the nanotubes can be effectively controlled (from 9.3 nm to 43.5 nm). Importantly, compared to the MoS_2_ nanofilm, the photoelectric response performance of the MoS_2_ nanotube arrays shows a significant improvement. When the wall thickness of the MoS_2_ nanotube is 25.2 nm, the photoelectric response performance is optimal, with a 2.8-fold increase in the on/off ratio and a 3.8-fold increase in the photoelectric response rate, mainly due to the significantly increased specific surface area and ordered nanoarray structure. These results indicate the great application potential of the as-prepared MoS_2_ nanotube nanoarrays on macroscale fabrication of low-cost and high-performance optoelectronic devices.

## Data Availability

The original contributions presented in this study are included in the article. Further inquiries can be directed to the corresponding author.
